# Clinical characteristics, prognostic factors, and predictive model for elderly primary spinal tumor patients who are difficult to tolerate surgery or refuse surgery

**DOI:** 10.3389/fonc.2022.991599

**Published:** 2022-11-10

**Authors:** Zhangheng Huang, Zhen Zhao, Yu Wang, Ye Wu, Chuan Guo, Qingquan Kong

**Affiliations:** Department of Orthopedics, Orthopedic Research Institute, West China Hospital, Sichuan University, Chengdu, Sichuan, China

**Keywords:** nomogram, primary spinal tumor, elderly, overall survival, SEER

## Abstract

**Background:**

As a rare tumor, surgery is the best treatment for primary spinal tumors. However, for elderly patients who cannot undergo surgery, the prognosis is often difficult to evaluate. The purpose of this study was to identify the risk factors that may lead to death and predict the prognosis of elderly patients with primary spinal tumors who have not undergone surgical treatment.

**Methods:**

In this study, 426 patients aged 60 years or older diagnosed with a primary spinal tumor between 1975 and 2015 were selected and included from the Surveillance, Epidemiology, and End Results database. A retrospective analysis was performed by using the Cox regression algorithm to identify independent prognostic factors. A nomogram model was developed based on the results. Multiple evaluation methods (calibration curve, receiver operating characteristic curve, and decision curve analyses) were used to evaluate and validate the performance of the nomogram.

**Results:**

A nomogram was developed, with age, histological type, and stage as independent prognostic factors. The results indicated that the prognostic risk tended to increase significantly with age and tumor spread. Osteosarcoma was found to have the most prominent risk prognosis in this patient group, followed by chondrosarcoma and chordoma. The area under the curve and the C-index of the model were both close to or greater than 0.7, which proved the high-differentiation ability of the model. The calibration curve and decision curve analyses showed that the model had high predictive accuracy and application value.

**Conclusions:**

We successfully established a practical nomogram to assess the prognosis of elderly patients with primary spinal tumors who have not undergone surgical treatment, providing a scientific basis for clinical management.

## Introduction

Primary spinal tumors, defined as the primary malignant tumors originating from the vertebral column and sacrum, are rare and account for less than 5% of all bone tumors and only about 0.2% of all tumors (1). As reported, the most common primary spinal tumors are osteosarcoma, chondrosarcoma, Ewing’s sarcoma, chordoma, and fibrosarcoma (2, 3). Because of the low incidence of neurological symptoms in the early stages, diagnosis is usually late. The incidence of malignant primary spinal tumors is highest in the age group of 41–60 years, accounting for 44%, whereas older adults over the age of 60 years account for only about 10% of the population with a predominance of malignant tumors (4). This result explains, to some extent, that older adults over the age of 60 years are often overlooked by researchers as a minority group in the population of patients diagnosed with primary spinal tumors (5). At the same time, because of the aggressiveness of the tumor to the surrounding tissues (nerves, blood vessels, etc.) and to avoid metastasis of cancer cells, the tumor must be removed according to the degree of integration with the spine when conditions permit, avoiding secondary recurrence (6). However, many elderly patients refuse surgery in favor of more conservative treatment options because of financial or personal physical reasons that make it difficult to tolerate surgery, which has led to the neglect of this population by clinical researchers.

A nomogram, a simple visualization of Cox regression results, can help clinicians predict the prognosis with significant advantages and greatly improve predictive accuracy (7). In a previous study, Zhou et al. constructed a prognostic nomogram model for patients with all types of primary spinal tumors (8). However, this nomogram was constructed for all ages, so the specificity and predictive accuracy of this model is questionable when applied to elderly primary spinal tumor patients who have not undergone surgical treatment. The Surveillance, Epidemiology, and End Results (SEER) database, established by the National Institutes of Research in 1973, is one of the most representative tumor database centers. Its huge volume of data can provide rich and reliable data to support our study (9, 10). In general, our study aimed to visualize the clinical analysis by the SEER database and construct a mathematical model. The model can make predictions on the overall survival of elderly patients with primary spinal tumors who have not undergone surgical treatment, providing a basis for clinical evaluation and exploring the best treatment.

## Materials and methods

### The patient’s data

In this retrospective study, we obtained data related to 426 patients aged 60 years or older. They were all diagnosed with primary spinal tumors between 1975 and 2015 through the SEER program. We do not require ethical review because the publicly available data cannot identify individuals. The criteria for inclusion and exclusion of data are as follows: (1) the patient’s primary tumor was located in the spine, (2) the patient did not undergo resection of the primary tumor, and (3) the patient’s age is greater than or equal to 60 years.

The personal information and clinical data of patients we extracted include age, sex, race, marital status, primary site, year of diagnosis, histological type, stage, receiving radiotherapy or not, receiving chemotherapy or not, survival time, and survival status. According to laboratory tests, as well as a clinical judgment, the period of disease was classified as follows: (1) localized (tumor confined to the periosteum), (2) regional (tumor extending from outside the periosteum to surrounding tissues), and (3) having distant metastases. Because the patients we included were older than 60 years, patients’ ages were divided into two groups (60–73 and ≥74 years). The patients’ races were divided into three groups: black, white, and other. The patients were split into five groups according to the time of diagnosis starting in 1970 and ending in 2010, every decade. The tumor was specified by the SEER variable “ICD/O/3 Hist-Behav,” including osteosarcoma, chondrosarcoma, chordoma, and others. The overall survival time is defined as the time from the date of diagnosis to the date of death or last follow-up.

### Statistical analysis

Accessing the SEER database *via* stat software to obtain data on elderly patients with primary spinal tumors who did not receive surgery and who met inclusion. We randomly divided all cases in a certain proportion into two cohorts for model construction and model validation. In the training cohort, we used the Cox regression algorithm to screen prognostic factors associated with the prognosis of elderly patients with unoperated spinal tumors. To assess the predictive accuracy and utility of the model, we depicted the calibration curves of the model in two cohorts and performed a decision curve analysis for both cohorts. Also, the area under the receiver operating characteristic curves for both cohorts was used to evaluate the discriminatory ability of the model. We also compared the difference in the accuracy of overall survival prediction using predictive models and individual prognostic factors. To further distinguish patients with high and low mortality risk, we made a further innovation based on the nomogram. We also constructed a relevant mortality risk classification system. In this study, all statistical analyses were performed by using SPSS (25.0); when the p-value is less than 0.05, we consider the existence of statistical significance.

## Results

### Characteristics of patients

According to our criteria, there were 426 eligible patients aged 60 years and older with a diagnosis of primary spinal tumor in the SEER database. The patients were grouped by age, and the grouping principle is shown in **Table 1**; we randomized all patients in a ratio of 7:3 to the training cohort (n = 300) and the validation cohort (n = 126). In the training cohort, 82.7% of patients had a primary site in the sacrum, 55.3% received radiotherapy, 25.3% received chemotherapy, and 35.3% had distal metastases. The patients in the validation cohort had similar proportions. The demographic and pathological characteristics of all included patients are demonstrated in **Table 1**.

**Table 1:** Clinical and pathological characteristics of elderly patients with primary spinal tumors who have not undergone surgical treatment.

Variables	SEER training cohort		SEER validation cohort	
	N = 300		N = 126	
	n	％	n	％
Age (years)				
60–73	131	43.7	50	39.7
≥74	169	56.3	76	60.3
Race				
Black	22	7.3	8	6.3
White	251	83.7	107	84.9
Other	27	9.0	11	8.7
Sex				
Male	169	56.3	73	57.9
Female	131	43.7	53	42.1
Year of diagnosis				
1970s	14	4.7	9	7.1
1980s	31	10.3	12	9.5
1990s	35	11.7	16	12.7
2000s	110	36.7	53	42.1
2010s	110	36.7	36	28.6
Histological type				
Osteosarcoma	86	28.7	38	30.2
Chondrosarcoma	73	24.3	36	28.6
Chordoma	77	25.7	31	24.6
Others	64	21.3	21	16.7
Primary site				
Vertebral column	52	17.3	27	21.4
Sacrum	248	82.7	99	78.6
Radiotherapy				
No	134	44.7	58	46.0
Yes	166	55.3	68	54.0
Chemotherapy				
No	224	74.7	101	80.2
Yes	76	25.3	25	19.8
Stage				
Localized	98	32.7	44	34.9
Regional	96	32.0	43	34.1
Distant	106	35.3	39	31.0
Marital status				
Unmarried	123	41.0	43	34.1
Married	177	59.0	83	65.9

### Visualization of Cox proportional hazards regression analyses

We used univariate and multivariate Cox proportional risk regression analyses to screen the prognostic risk factors of the included patients. The univariate Cox analysis indicated that age, stage, race, and histological type were associated factors. The above variables with p values of less than 0.05 in the results of the univariate analysis were included in the multivariate analysis. The results also showed that age, histological type, and stage were independent prognostic factors affecting elderly primary spinal tumor patients who refused surgery, and these three variables were ultimately used to construct the nomogram (**Table 2**).

**Table 2:** Analysis of univariate and multivariate Cox regression in elderly patients with primary spinal tumors who have not undergone surgical treatment.

Characteristics	Univariate analysis		Multivariate analysis	
	HR (95% CI) P value		HR (95% CI) P value	
Age				
60-73	Reference		Reference	
≥74	1.291 (1.011–1.648)	0.041	1.535 (1.190–1.981)	0.001
Race				
Black	Reference	0.066		
White	0.738 (0.471–1.157)	0.186		
Other	0.493 (0.270–0.899)	0.021		
Sex				
Male	Reference			
Female	0.849 (0.666–1.081)	0.185		
Year of diagnosis				
1970s	Reference	0.132		
1980s	1.268 (0.674-2.388)	0.461		
1990s	0.815 (0.436-1.522)	0.521		
2000s	0.768 (0.438-1.347)	0.357		
2010s	0.771 (0.438-1.359)	0.369		
Histological type				
Osteosarcoma	Reference		Reference	
Chondrosarcoma	0.602 (0.432-0.838)	0.003	0.601 (0.430-0.840)	0.003
Chordoma	0.290 (0.205-0.411)	<0.001	0.318 (0.218-0.464)	<0.001
Others	0.752 (0.538-1.052)	0.096	0.809 (0.576-1.138)	0.224
Radiotherapy				
No	Reference			
Yes	0.965 (0.757-1.229)	0.771		
Chemotherapy				
No	Reference			
Yes	1.140 (0.864-1.503)	0.355		
Stage				
Localized	Reference		Reference	0.001
Regional	1.292 (0.950-1.758)	0.103	1.186 (0.870-1.617)	0.281
Distant	2.338 (1.732-3.156)	<0.001	1.817 (1.325-2.491)	<0.001
Marital status				
Unmarried	Reference			
Married	1.089 (0.853-1.389)	0.495		

### Construction of nomogram

**Figure 1:**
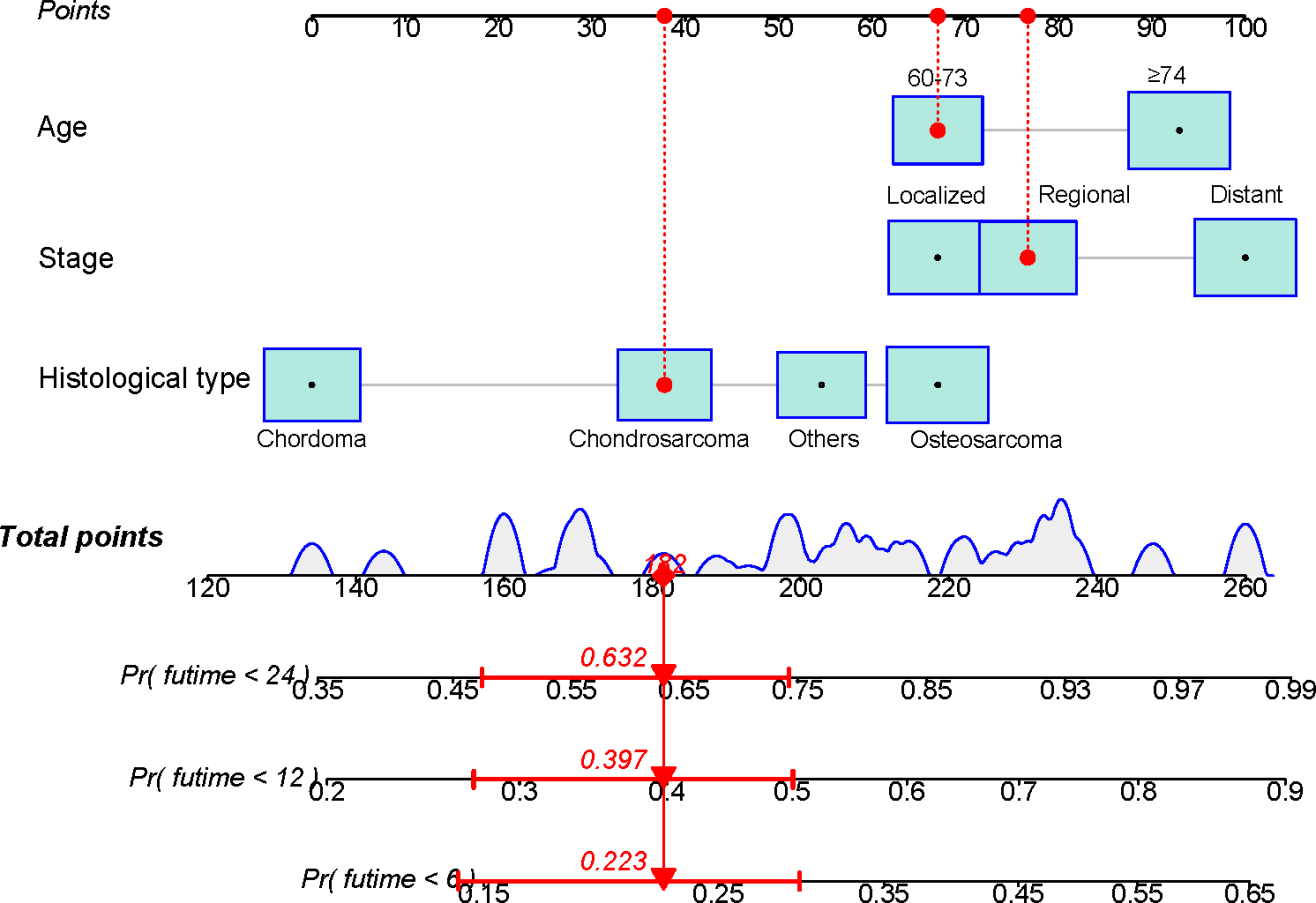
A nomogram for predicting the overall survival in patients with elderly patients with primary spinal tumors who have not undergone surgical treatment.

Based on the independent risk variables obtained from the Cox analysis, we created a nomogram for elderly patients with primary spinal tumors who did not receive surgery (**Figure 1**). We can find the corresponding points on the horizontal axis (time axis) by summing the scores corresponding to each variable of the patient to be predicted as the total score. From there, we can predict the patient’s overall survival probability at a particular point in time.

### Validation of nomogram

**Figure 2:**
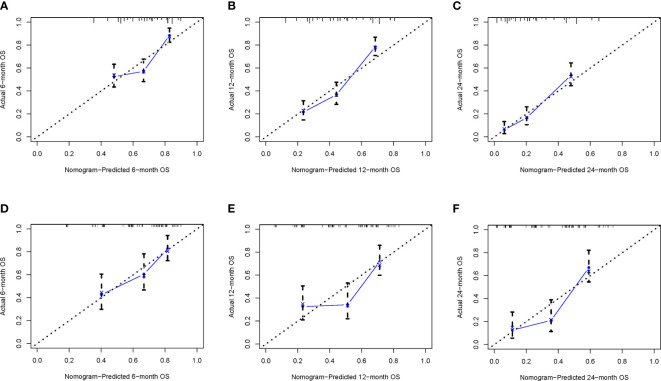
The calibration curves of the nomogram are depicted in the training cohort **(A–C)** and the validation cohort **(D–F)**.

**Figure 3:**
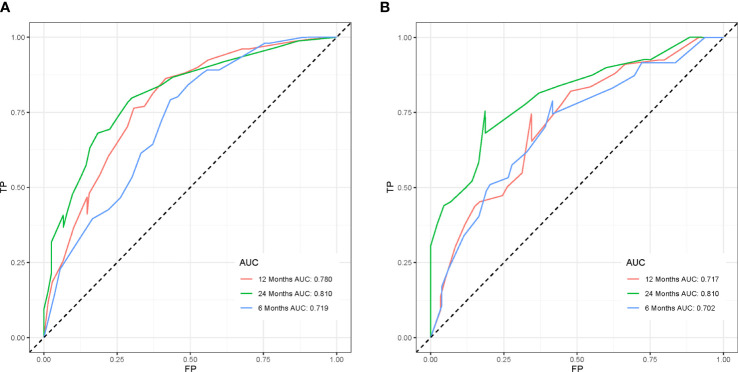
The receiver operating characteristic curves of the nomogram model is depicted in the training cohort **(A)** and the validation cohort **(B)**.

**Figure 4:**
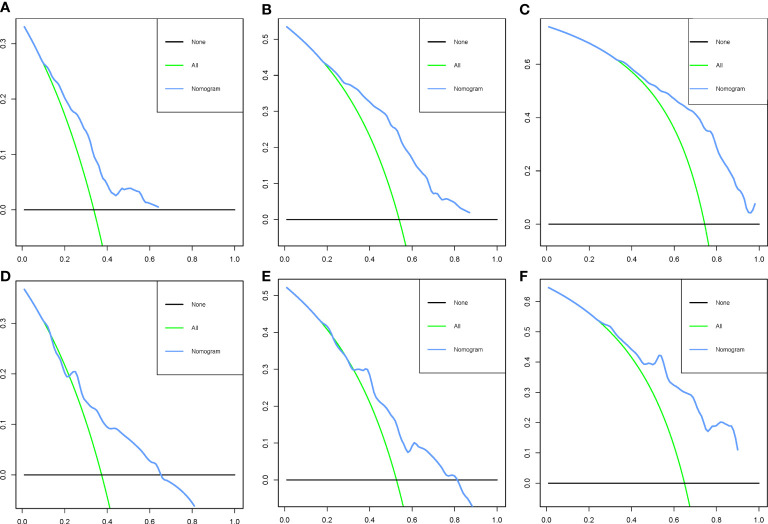
Decision curve analysis of the nomogram in the training cohort **(A–C)** and the validation cohort **(D–F)**.

To ensure the accuracy of the model, we resampled the training and validation cohorts, and the results showed a C-index of 0.685 in the training cohort and 0.681 in the validation cohort, which proved the predictive reliability of our nomogram. Moreover, we found that the predicted survival probabilities of the validation cohort and the training cohort of the nomogram were in fairly high agreement with the observed survival probabilities (**Figure 2**), which both validated the clinical predictive assessment value of this nomogram. The area under the curve for both cohorts is greater than 0.7, further demonstrating the high differentiation ability of the model (**Figure 3**). Over a wide range of mortality risks, the decision curve analysis showed that the nomogram resulted in greater net benefits, suggesting that columnar maps have good clinical efficacy (**Figure 4**).

### Definition of the risk classification system

**Figure 5:**
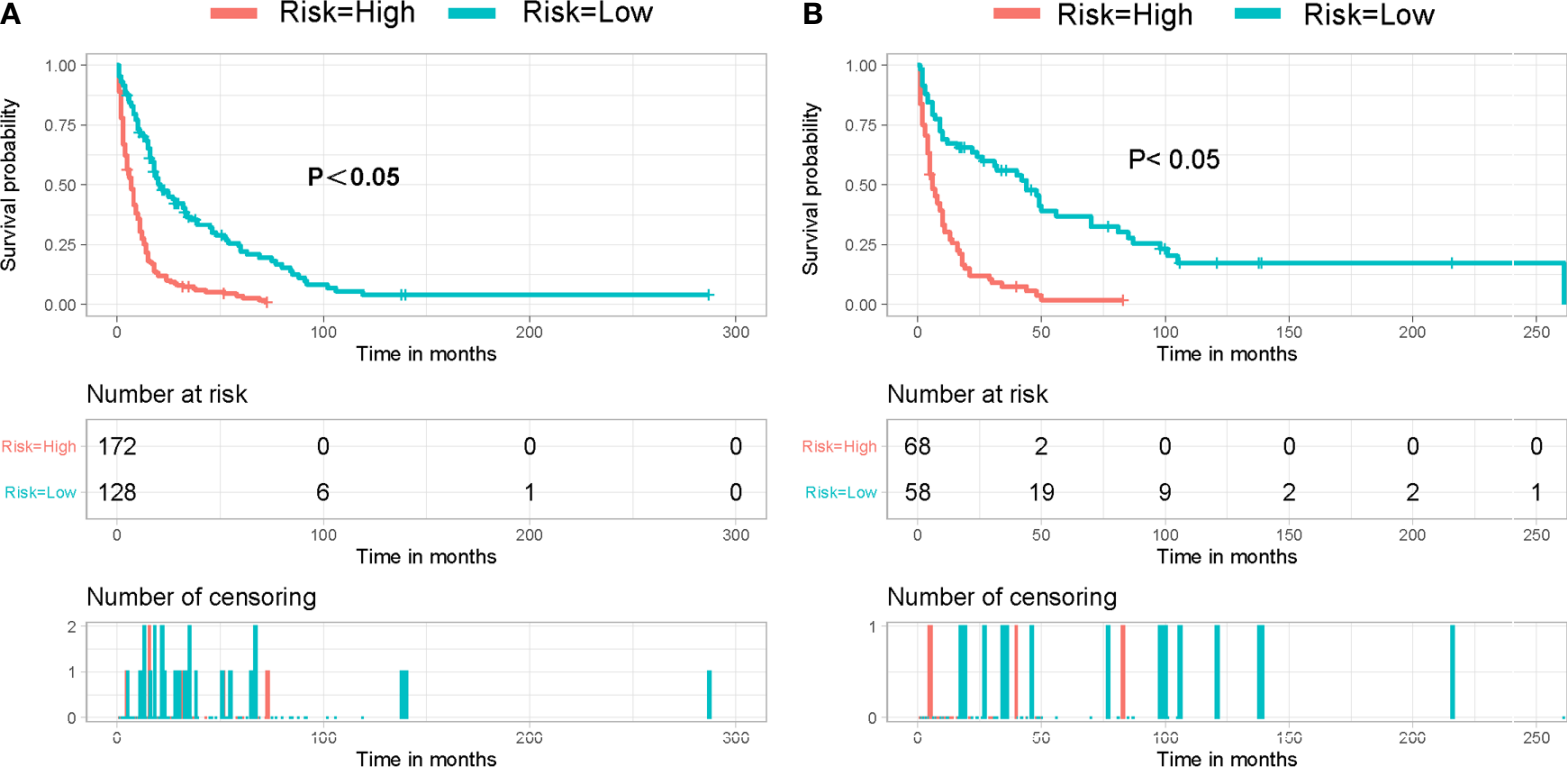
Kaplan–Meier survival curve for patients in different risk groups in both cohorts: **(A)** training cohort and **(B)** validation cohort.

Based on the patient scores calculated by nomogram, we further divided all patients between the training cohort and the validation cohort into a high-risk group (total score < 201) and a low-risk group (total score ≥ 201). By fitting Kaplan–Meier survival curves, we found significant prognostic differences between patients in the high-risk group and those in the low-risk group in both cohorts (**Figure 5**).

## Discussion

As a relatively rare disease, surgery remains the best choice of treatment for primary spinal tumors (1, 6, 11). Elderly patients, as a minority group of patients, also have a high probability of refusing surgical treatment for various reasons. Because almost all studies have focused on the evaluation of surgical treatment in patients with primary spinal tumors, the prognostic assessment of elderly patients who have not undergone surgical treatment is a challenge for clinicians without relevant experience. In contrast to previous studies, our focus is on the group of elderly patients with primary spinal tumors who have not undergone surgery. Our study successfully established a prognostic model in elderly patients with primary spinal tumors based on the SEER database. We also completed a better validation fit, which identified age, histological type, and stage as important prognostic influencing factors while excluding irrelevant factors (race, gender, and radiotherapy status) in the group of elderly patients treated non-operatively, providing a powerful aid to clinical diagnosis and treatment.

There is now sufficient evidence that age is a significant negative factor in the prognosis of primary spinal tumors, with some studies showing an increased risk probability of death in patients 55 years and older, which is consistent with our results (12). Interestingly, however, there was no significant difference in survival between patients younger than 56 years and 56–72 years in the study by Qiang Zhou (13). This differs from the results of our study, which may be related to the criteria of the patients we included, and whether or not they underwent surgery may be an important reason. In addition, the metastatic nature of primary spinal tumors has been identified as the most significant poor prognostic factor in previous studies, with lung, bone, and bone marrow as the most susceptible tissues and organs more prone to tumor metastasis (14–16). The probability of such metastasis shows a strong positive correlation with the delayed disease stage, whereas the changes in the body’s internal environment caused by aging can also greatly increase the invasive capacity of tumor cells (17). Therefore, we suggested that age and disease stage act as independent prognostic influences while they are also related to each other.

On the other hand, immediate postoperative radiation therapy for chordoma and chondrosarcoma was found to significantly prolong local progression-free time and overall survival in a previous study (18). The National Comprehensive Cancer Network for Bone Cancer screening guidelines also clearly state the significant role of neoadjuvant and adjuvant multidrug chemotherapy in improving the prognosis of patients with osteosarcoma and Ewing’s sarcoma (16). This conclusion is somewhat similar to the study we did previously (7). However, radiation therapy and chemotherapy were not included as statistically significant variables in our study in the nomogram. Chemotherapy was defined as an unfavorable factor affecting patient prognosis in the population of patients with older age and distant metastases in a study by Lei (8). Furthermore, previous studies have also proved that radiotherapy has different effects on different histological types of tumors. For example, chondrosarcoma is often treated with radiation, whereas neither radiation nor chemotherapy has a significant effect on chordoma (8, 16, 19). Therefore, we believe that, in addition to the crucial influence of surgical intervention, the physical condition of the elderly patient and the histological nature of the tumor in terms of tolerance to chemotherapy and radiotherapy should also be taken into account. Whether radiotherapy and chemotherapy can achieve the desired results in the context of an elderly patient population removed from surgery requires more research.

The three independent prognostic factors we identified (age, histological type, and stage) have been shown in previous studies to be important prognostic influences in patients with primary spinal tumors (13, 20–23). However, no investigator has indicated whether it has prognostic significance for assessment in the elderly patient population. The acceptance of surgical treatment as an important prerequisite was shown by us in this study to greatly influence a variety of prognostic factors. Therefore, our prognostic risk factor model based on the SEER database for elderly patients is of great clinical significance. In clinical practice, physicians can include patients’ risk factors in the nomogram to evaluate their prognosis and select the most suitable treatment such as conservative, aggressive, or palliative care. Based on the results of the nomogram, we can distinguish patients with a high risk of death. At the same time, we can adjust the strategy of treatment in time and increase the attention to such patients. Finally, we must acknowledge the limitations of our study, as we did not perform a comparison of validation cohorts, and a single database could have caused bias in the results. Despite its shortcomings, our study is the first to use elderly patients with primary spinal tumors who have not received surgical treatment as subjects and to achieve more groundbreaking findings.

## Conclusions

In summary, our study proves that patients’ age at diagnosis, tumor histological type, and stage are independent prognostic factors affecting elderly patients with primary spinal tumors who refused surgery. These three variables were incorporated to construct the first nomogram to predict the overall survival probability. Clinicians can use the nomogram constructed in this study as a valid and convenient assessment tool to perform personalized survival assessments and identify the risk of death in elderly patients with primary spinal tumors who have not undergone surgery.

## Data availability statement

Publicly available datasets were analyzed in this study. This data can be found here: https://seer.cancer.gov/seerstat/.

## Ethics statement

This study is based on data from public databases and therefore does not require informed consent from patients.

## Author contributions

ZH and QK conceived and designed the study. ZH and ZZ performed the literature search. ZH, YuW, and YeW generated the figures and tables. ZH, ZZ, and CG analyzed the data. ZH and ZZ wrote the manuscript, and QK critically reviewed the manuscript. ZH and QK supervised the research. All authors have read and approved the manuscript.

## Funding

This work was supported by the Sichuan Science and Technology Program (2020YFS0080, 2020YFQ0007, and 2021JDRC0159), the Science and Technology Project of Tibet Autonomous Region (XZ201901-GB-08), the National Natural Science Foundation of China (81171731), and the 1·3·5 Project for Disciplines of Excellence, West China Hospital, Sichuan University (ZYJC21026 and ZYJC21077).

## Conflict of interest

The authors declare that the research was conducted in the absence of any commercial or financial relationships that could be construed as a potential conflict of interest.

## Publisher’s note

All claims expressed in this article are solely those of the authors and do not necessarily represent those of their affiliated organizations, or those of the publisher, the editors and the reviewers. Any product that may be evaluated in this article, or claim that may be made by its manufacturer, is not guaranteed or endorsed by the publisher.

## Abbreviations

SEER, Surveillance, Epidemiology, and End Results.
